# Supplementary material for: Identification of photoperiod-induced specific miRNAs in the adrenal glands of Sunite sheep (Ovis aries)
Source: Front Vet Sci. 2022 Jul 22;9:888207. doi: 10.3389/fvets.2022.888207 (PMC9354845; doi:10.3389/fvets.2022.888207)
Supplement: Supplementary file 1 [file Data_Sheet_1.ZIP › Supplementary file/miRDeep2 file.zip!/miRDeep2 file/Know_Structure/SP42A2/oar-mir-374b.pdf]

miRBase precursor : oar-mir-374b
Total read count : 30700
oar-miR-374b read count : 30216
remaining reads : 484

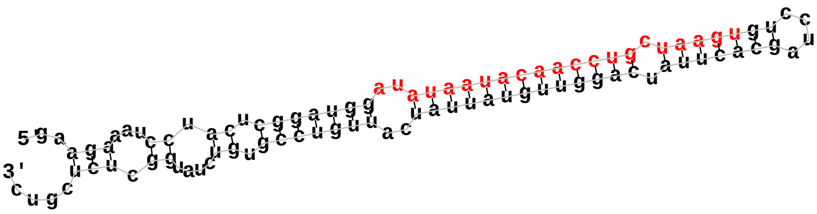

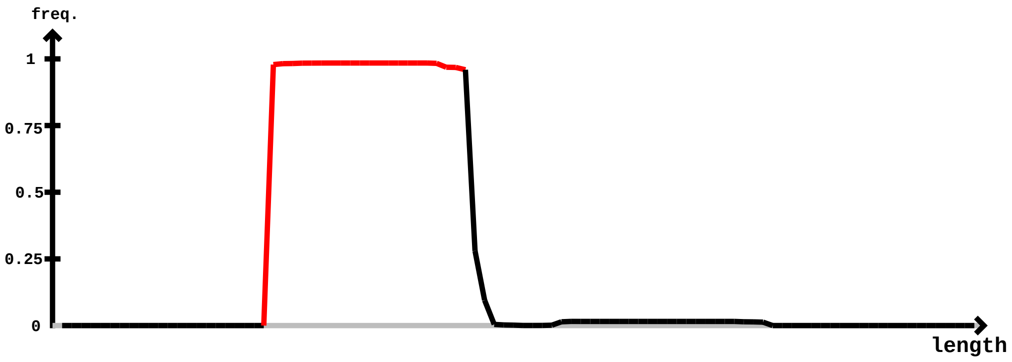

oar-miR-374b

```
5'- gaagaaauccuacucggaugguauauaauacaaccugcuaagu
```

```
5'- gaagaaauccuacucggauggauauaauacaaccugcuaaguguccuagcacuuaucagguuguauuaucauuguccgugucuauggcucucguc   -3'    exp
    ((((..((...((.(((((((..(((((((((((((.(((((((....))))))).)))))))))))))..))))))).))..))..)).))...    reads    mm    sample
    .....................auauaauacaaccuAcu.........................................................    1        1     seq
    .....................auauaauacaaccugcA.........................................................    4        1     seq
    .....................auauaauUcaaccugcu.........................................................    1        1     seq
    .....................auauGauacaaccugcu.........................................................    1        1     seq
    .....................auauaCuacaaccugcu.........................................................    1        1     seq
    .....................Cuauaauacaaccugcu.........................................................    3        1     seq
    .....................auauaauacaaccugAu.........................................................    1        1     seq
    .....................auauaauacaaccugcu.........................................................    30       0     seq
    .....................auauaauacaaccugcua........................................................    443      0     seq
    .....................auauaauacaaccugcuaa.......................................................    25       0     seq
    .....................auauaauacaaccugcuaag......................................................    266      0     seq
    .....................auauaauacaaccugcuaagu.....................................................    20816    0     seq
    .....................auauaauacaaccugcuaagug....................................................    5680     0     seq
    .....................auauaauacaaccugcuaagugu...................................................    2714     0     seq
    .....................auauaauacaaccugcuaaguguc..................................................    47       0     seq
    .....................auauaauacaaccugcuaagugucc.................................................    7        0     seq
    .....................auauaauacaaccugcuaaguguccu................................................    2        0     seq
    .....................auauaauacaaccugcuaaguguccuagcacu..........................................    1        0     seq
    ......................uauaauacaaccugcua........................................................    2        0     seq
    ......................uauaauacaaccugcuaagu.....................................................    24       0     seq
    ......................uauaauacaaccugcuaagug....................................................    8        0     seq
    ......................uauaauacaaccugcuaagugu...................................................    59       0     seq
    ......................uauaauacaaccugcuaaguguc..................................................    8        0     seq
    ......................uauaauacaaccugcuaagugucc.................................................    3        0     seq
    ......................uauaauacaaccugcuaaguguccu................................................    3        0     seq
    .......................auaauacaaccugcuaagu.....................................................    6        0     seq
    .......................auaauacaaccugcuaagugu...................................................    3        0     seq
    .......................auaauacaaccugcuaaguguc..................................................    4        0     seq
    .......................auaauacaaccugcuaagugucc.................................................    2        0     seq
    .......................auaauacaaccugcuaaguguccu................................................    2        0     seq
    ........................uaauacaaccugcuaagu.....................................................    1        0     seq
    ........................uaauacaaccugcuaagugu...................................................    2        0     seq
    ........................uaauacaaccugcuaaguguc..................................................    4        0     seq
    ........................uaauacaaccugcuaagugucc.................................................    9        0     seq
    ........................uaauacaaccugcuaaguguccu................................................    26       0     seq
```

oar-miR-374b

```
gaagaaauccuacucggauggauauaauacaaccugcuaaguguccuagcacuuaucagguuguauuaucauuguccgugucuauggcucucguc
........................uaauacaaccugcuaaguguccuagcacuu.........................................    1     0    seq
.........................aauacaaccugcuaagugucc.................................................    2     0    seq
.........................aauacaaccugcuaaguguccua...............................................    1     0    seq
.........................aauacaaccugcuaaguguccuagc.............................................    2     0    seq
..........................auacaaccugcuaaguguccu................................................    5     0    seq
..........................auacaaccugcuaaguguccuag..............................................    1     0    seq
..........................auacaaccugcuaaguguccuagcacuu.........................................    1     0    seq
...........................uacaaccugcuaaguguccu................................................    1     0    seq
...........................uacaaccugcuaaguguccuagc.............................................    1     0    seq
.............................caaccugcuaaguguccuagcacu..........................................    1     0    seq
...............................................agcacuuaucagguuguauuauc.........................    1     0    seq
................................................gcacuuaucagguuguauuauc.........................    1     0    seq
.................................................cacuuaucagguuguauuauc.........................    1     0    seq
.................................................cacuuaucagguuguauuauca........................    2     0    seq
..................................................Ccuuaucagguuguauuauc.........................    2     1    seq
..................................................acuuaucagguuguauuauc.........................    22    0    seq
..................................................acuuaucagguuguauuauca........................    4     0    seq
..................................................acuuaucagguuguauuaucau.......................    5     0    seq
..................................................acuuaucagguuguauuaucauu......................    1     0    seq
...................................................cuuaucagguuguauuauc.........................    13    0    seq
...................................................cuuaucagguuguauuaucU........................    1     1    seq
...................................................cuuaucagguuguauuauca........................    5     0    seq
...................................................cuuaucagguuguauuauAau.......................    1     1    seq
...................................................cuuaucagguuguauuaucau.......................    20    0    seq
...................................................cuuaucagguuguauuaucauu......................    351   0    seq
....................................................uuaucagguuguauuaucau.......................    3     0    seq
....................................................uuaucagguuguauuaucauu......................    42    0    seq
```
